# Alcohol screening a brief intervention: a self-paced program for nurses

**DOI:** 10.1186/1940-0640-10-S2-O18

**Published:** 2015-09-24

**Authors:** Deborah Finnell, Ann M Mitchell, Christine L Savage, Irene Kane, Robert Kearns, Nathan Poole, Hilda Rizzo-Busack, Scott Coulson

**Affiliations:** 1 Johns Hopkins School of Nursing, Baltimore, MD, USA; 2 University of Pittsburgh School of Nursing, Pittsburgh, PA, USA

## Background

Nurses are in key positions to plan and implement alcohol screening and brief intervention (aSBI). To simultaneously advance aSBI of nurses in various roles, we developed a self-paced program for Clinical Nurse Leaders, Nurse Informaticists, Nurse Administrators, and Registered Nurses including Advanced Practice Nurses. The content for the program is based on the Centers for Disease Control & Prevention (CDC) 2014 Planning and Implementation Screening and Brief Intervention for Risky Alcohol Use: A Step-by-Step Guide for Primary Care Practices. The objective is to present the results of a cooperative agreement between the Centers for Disease Control & Prevention, Johns Hopkins School of Nursing, and the University of Pittsburgh School of Nursing. The framework used to design the aSBI Program and key aspects of the program will be demonstrated and discussed.

## Material and methods

The aSBI Program flow, depicted in the CDC guide defined the modules for learning: patient population, assess alcohol consumption, negative screen (and subsequent conversation), positive screen (and assessment of harm and dependence), brief intervention and referral to treatment. The modules were developed using Articulate Storyline, the premier rapid e-learning development platform, and hosted on a learning management system. Special focus was given to the assessment portion of the e-learning modules to ensure learners are provided with authentic assessments that accurately measure their mastery of real-world skills needed to be successful when applying the module educational materials.

## Results

When finalized, the self-paced aSBI Program, funded by the American Association of Colleges of Nursing through the CDC, will be widely disseminated to the nursing community across the U.S.

## Conclusions

This easily accessible on-line educational program will bring evidence-based alcohol screening and intervention to current and future nurses.

## References

[B1] Centers for Disease Control and Prevention Planning and implementing screening and brief intervention for risky alcohol use: A step-by-step guide for primary care practices 2014 Atlanta, GA: Center for Disease Control and Prevention, National Center on Birth Defects and Developmental Disabilities

